# Human hepatic lipase overexpression in mice induces hepatic steatosis and obesity through promoting hepatic lipogenesis and white adipose tissue lipolysis and fatty acid uptake

**DOI:** 10.1371/journal.pone.0189834

**Published:** 2017-12-15

**Authors:** Lídia Cedó, David Santos, Núria Roglans, Josep Julve, Victor Pallarès, Andrea Rivas-Urbina, Vicenta Llorente-Cortes, Joan Carles Laguna, Francisco Blanco-Vaca, Joan Carles Escolà-Gil

**Affiliations:** 1 Institut d’Investigacions Biomèdiques (IIB) Sant Pau, Barcelona, Spain; 2 CIBER de Diabetes y Enfermedades Metabólicas Asociadas, CIBERDEM, Hospitalet de Llobregat, Spain; 3 Department of Pharmacology and Therapeutic Chemistry, School of Pharmacy, University of Barcelona, Barcelona, Spain; 4 Departament de Bioquímica, Biología Molecular i Biomedicina, Universitat Autònoma de Barcelona, Barcelona, Spain; 5 Lipids and Cardiovascular Pathology Group. CSIC-ICCC-IIB-Sant Pau and Instituto de Investigaciones Biomédicas de Barcelona (IibB)-CSIC, Barcelona, Spain; 6 Centro de Investigación Biomédica en Red de Enfermedades Cardiovasculares, CIBERCV, Madrid, Spain; State University of Rio de Janeiro, BRAZIL

## Abstract

Human hepatic lipase (hHL) is mainly localized on the hepatocyte cell surface where it hydrolyzes lipids from remnant lipoproteins and high density lipoproteins and promotes their hepatic selective uptake. Furthermore, hepatic lipase (HL) is closely associated with obesity in multiple studies. Therefore, HL may play a key role on lipid homeostasis in liver and white adipose tissue (WAT). In the present study, we aimed to evaluate the effects of hHL expression on hepatic and white adipose triglyceride metabolism *in vivo*. Experiments were carried out in hHL transgenic and wild-type mice fed a Western-type diet. Triglyceride metabolism studies included β-oxidation and *de novo* lipogenesis in liver and WAT, hepatic triglyceride secretion, and adipose lipoprotein lipase (LPL)-mediated free fatty acid (FFA) lipolysis and influx. The expression of hHL promoted hepatic triglyceride accumulation and *de novo* lipogenesis without affecting triglyceride secretion, and this was associated with an upregulation of *Srebf1* as well as the main genes controlling the synthesis of fatty acids. Transgenic mice also exhibited more adiposity and an increased LPL-mediated FFA influx into the WAT without affecting glucose tolerance. Our results demonstrate that hHL promoted hepatic steatosis in mice mainly by upregulating *de novo* lipogenesis. HL also upregulated WAT LPL and promoted triglyceride-rich lipoprotein hydrolysis and adipose FFA uptake. These data support the important role of hHL in regulating hepatic lipid homeostasis and confirm the broad cardiometabolic role of HL.

## Introduction

Hepatic lipase (HL) is a lipase mainly secreted from the liver and localized mainly on the surface of liver sinusoidal capillaries [1]. HL exhibits phospholipase A1 and triglyceride hydrolase activities, thereby hydrolyzing the phospholipids and triglycerides of intermediate density, low density lipoproteins (LDL) of large size and high density lipoproteins (HDL), and releasing free fatty acids (FFA) and smaller lipoprotein particles [1, 2]. Beyond its lipolytic actions, HL may also facilitate the selective uptake of HDL cholesteryl esters and the removal of apoB-containing lipoprotein remnants via promoting their binding to cell-surface heparin sulphate proteoglycans [1, 2].

Excessive intrahepatic triglyceride content, also termed steatosis, is the main feature of nonalcoholic fatty liver disease (NAFLD), which occurs when the rate of hepatic fatty acid uptake and *de novo* triglyceride synthesis are higher than the rate of fatty acid oxidation and hepatic triglyceride secretion [3, 4]. Hepatic steatosis is directly related to enhanced oxidative stress and lipotoxicity induced by excess fatty acids at the intracellular level [5]. Obesity is also associated with the risk of NAFLD [6]. A significant association between HL and obesity has been found in several human reports. Indeed, increased HL activity correlated with increased intra-abdominal fat in premenopausal women [7, 8], whereas reduced accumulation of intra-abdominal fat was also correlated with reduced HL activity in men undergoing diet-induced weight loss [9]. Furthermore, HL activity positively correlated with the severity of steatosis in NAFLD patients, even after correcting for body mass index and homeostasis model assessment (HOMA) for the insulin resistance index [10]. Importantly, both liver HL activity and the amount of hepatic lipids were significantly reduced in morbidly obese subjects after bariatric surgery [11].

Genetically-engineered mice for mouse HL have been used for investigating the effects of HL deficiency on obesity and hepatic triglyceride accumulation [12–14]. Indeed, HL deficiency prevented diet-induced obesity and steatosis without affecting glucose homeostasis [12]. Nevertheless, in another study, HL deficiency promoted steatosis, inflammation, and glucose intolerance [13]. It should be noted that mouse HL has a lower affinity for heparin sulfate polysaccharides compared with that of human HL (hHL). Therefore, the mouse HL is mainly found free in the circulation, whereas the human HL mainly remains anchored to liver proteoglycans [15]. A recent report showed that the expression of the catalytic activity of hHL was able to reverse the lean phenotype of the former HL-deficient mice [14]. This rescue of body fat gain seemed to be related to a reduced energy expenditure [14]. However, the specific pathways through which HL regulates triglyceride and fatty acid metabolisms remain largely unknown.

This study aimed to evaluate the effects of hHL on hepatic and adipose tissue triglyceride metabolic pathways *in vivo*. We show that overexpression of hHL in mice promoted hepatic triglyceride accumulation mainly by upregulating the hepatic lipogenesis without affecting hepatic triglyceride secretion. Furthermore, we also found that hHL expression upregulated adipose-specific lipoprotein lipase (LPL), thereby increasing adipose FFA influx.

## Materials and methods

### Mice and diet

Wild-type (WT) and hHL transgenic mice in the C57BL/6J background were obtained from Jackson Laboratories (#000664 and 003285, respectively, Bar Harbor, ME) [16]. The genotype of the offspring was confirmed by polymerase chain reaction (PCR) using the WT and targeted allele-specific primers recommended by Jackson Laboratories (https://www.jax.org/strain/003285). All animal procedures were conducted in accordance with published regulations and reviewed and approved by the Institutional Animal Care Committee of the Institut de Recerca of the Hospital de la Santa Creu i Sant Pau (Permit Number: 7083). Mice were kept in a temperature-controlled (22°C) room with a 12-hour light/dark cycle and food and water were provided *ad libitum*. For this study, we used eight- to nine-week-old male mice fed a Western-type diet (TD.88137, Harlan Teklad, Madison, WI, containing 21% fat and 0.2% cholesterol) for 16 weeks. Body weight and food intake were monitored. For studying triglyceride metabolism under postprandial conditions, mice were administered an oral fat gavage consisting of an intragastric load of 150 μL of olive oil and euthanized after 90 minutes (min), except for hepatic triglyceride secretion studies, which use an inhibitor of lipolysis; therefore, assays were carried out under fasting conditions to avoid the accumulation of intestinal triglyceride-rich lipoproteins (see 2.5 section). Blood was obtained by cardiac puncture and serum obtained by centrifugation at 10,000 g for 10 min at 4°C. Tissues were collected, weighted, and immediately frozen in liquid nitrogen for later use.

### Lipid analyses and enzyme activities

Serum triglycerides, FFA, total serum cholesterol and phospholipids, and HDL lipids were determined enzymatically using commercial kits adapted to a COBAS 6000 autoanalyzer (Roche Diagnostics, Rotkreuz, Switzerland). Triglyceride determinations were corrected for free-glycerol. Hepatic lipids were extracted with isopropyl alcohol-hexane (2:3, v:v) [17]. The lipid layer was collected, evaporated, and resuspended in cholate 0.5% (w:v). Hepatic triglycerides, total cholesterol, free-cholesterol, and phospholipids were determined using commercial kits adapted to the COBAS 6000 autoanalyzer.

Liver and white adipose tissue (WAT) homogenates were obtained in a Polytron homogenizer at 4°C with a small piece of tissue in buffer EDH pH 7.4 [1 mM EDTA, 1 mM DTT, 10 mM HEPES, 5 U/mL heparin, plus three protease inhibitors: 0.5 mM PMSF, 4 μg/mL aprotinin, and 4 μg/mL pepstatin). Homogenates were centrifuged at 4°C for 10 min at 1,000 g, and the clear supernatants were used in the enzyme assay. LPL and HL activities were measured in serum and tissues using a radiolabeled glycerol tri[9,10-(n)-^3^H]oleate emulsion (21.0 Ci/mmol; Perkin Elmer, Waltham, MA) [18]. LPL was inactive in the HL assay due to the high NaCl concentration [18].

### Histology

Formalin-fixed livers and epididymal WAT (eWAT) were embedded in paraffin and samples were sectioned at 5-μm for haematoxylin and eosin (H&E) staining [19]. Livers were embedded in OCT compound, and 10-μm sections were cut on a cryostat. Tissue sections were stained with Oil Red O for 2 min and counterstained with hematoxylin [20]. Adipocyte cross-sectional area was determined using Image J software (available at http://rsbweb.nih.gov/ij/).

### Determination of β-oxidation activity

Liver and eWAT were homogenized in a buffer composed of 150 mM NaCl, 1 mM dithiothreitol, 30 mM EDTA, and 50 mM KH_2_PO_4_. β-oxidation activity was determined with 30 μg of postnuclear supernatant by determining the conversion of palmitoyl CoA-1-^14^C into acetyl-CoA [21].

### *De novo* lipogenesis

Determination of *de novo* lipogenesis was performed after intraperitoneal (i.p.) injection of 10 μCi/g of ^3^H_2_O (Perkin Elmer, Waltham, MA). One hour later, animals were euthanized and blood and tissues obtained. The rate of incorporation of tritium from ^3^H_2_O into fatty acids in the liver and eWAT was then determined (adapted from [22]). Briefly, tissues were digested in 30% KOH at 70°C for 10 min and saponification was performed by adding ethanol (1:1, v:v) and incubating at 70°C for 2 hours. Fatty acids were released by neutralizing with H_2_SO_4_ 6M and extracted three times with 5 mL of petroleum ether. The petroleum ether extracts were dried and radioactivity was determined by liquid scintillation counting [22].

### *In vivo* triglyceride secretion rate

Mice were bled to measure baseline plasma triglyceride concentration under fasting conditions. Then, anesthetized mice were injected intravenously with Triton WR-1339 (Sigma-Aldrich, St. Louis, MO) at a dose of 500 mg/kg dissolved in a 15% solution of 0.9% NaCl, which inhibited lipolysis completely [23]. Blood was collected after the Triton injection and plasma triglycerides were measured. Triglyceride accumulation in the serums 60 and 120 min after injection was compared between mice of each genotype.

### *In vivo* triglyceride-rich lipoprotein lipolysis and FFA uptake

The triglyceride-rich lipoproteins (TRL) (density 1.006 g/mL) were isolated by ultracentrifugation from serum of WT mice with an analytical fixed-angle rotor (Beckman Coulter, Fullerton, CA, USA) and were radiolabeled with 40 μCi glycerol tri[9,10(n)-^3^H]oleate as previously described [24]. Each mouse was intravenously injected with 500,000 cpm of the TRL fraction in 100 μL of 0.9% NaCl and euthanized after 10 min. Radiolabeled liver and WAT triglycerides were separated from FFA using methanol:chloroform:heptane 1.4:1.25:1 (v:v:v) and 0.1 M H_3_BO_3_-KCO_3_ at pH 10.5 [24]. The radioactivity in the triglycerides and FFA fraction was determined by liquid scintillation counting.

### Glucose and insulin determination and glucose tolerance test

Serum glucose was determined enzymatically using a commercial kit adapted to a COBAS 6000 autoanalyzer (Roche Diagnostics, Rotkreuz, Switzerland) and serum insulin was assayed by ELISA (Mercodia, Uppsala, Sweden). The HOMA-IR index was calculated by multiplying the values of glucose (mM) and insulin (μU/mL) and dividing by 22.5. The glucose tolerance test was performed by i.p. administration of glucose (1.3 mg/g of body mass) [17]. After that, serum glucose was measured at t = 0, 15, 30, 60, 120, and 180 min. The area under the curve (AUC) was calculated [17].

### Quantitative real-time polymerase chain reaction analyses

Total RNA was extracted from liver and eWAT using TRIzol LS Reagent (Invitrogen, Carlsbad, CA) following the manufacturer’s instructions and purified using an RNeasy Plus Mini Kit (Qiagen, Hilden, Germany). cDNA was generated using Oligo(dT)_23_ and dNTPs mix (Sigma-Aldrich, St. Louis, MO) and M-MLV reverse transcriptase RNase H minus point mutant (Promega, Madison, WI) and it was subjected to quantitative real-time PCR amplification using TaqMan Master Mix (Promega, Madison, WI) and specific TaqMan probes (Applied Biosystems, Foster City, CA) for acetyl-Coenzyme A carboxylase α (*Acaca;* Mm01304257_m1), acyl-Coenzyme A oxidase 1 (*Acox1;* Mm01246831_m1), apolipoprotein a1 (*Apoa1*; Mm 00437569_m1), *Apob* (Mm01545159_m1), cluster of differentiation 36 (*Cd36;* Mm01135198_m1), carnitine palmitoyltransferase 1a *(Cpt1a;* Mm00550438_m1), diacylglycerol O-acyltransferase 1 (*Dgat1;* Mm00515643_m1), fatty acid synthase *(Fasn;* Mm01253292_m1), lecithin-cholesterol acyltransferase (*Lcat*; Mm00500505_m1), ldl receptor (*Ldlr*; Mm 00440169_m1), hormone-sensitive lipase expression (*Lipe*; Mm00495359_m1), lipoprotein lipase (*Lpl;* Mm00434764_m1), microsomal triglyceride transfer protein (*Mttp;* Mm00435015_m1), nuclear receptor subfamily 1, group H, member 3 (*Nr1h3;* Mm00443451_m1), peroxisome proliferator activated receptor α (*Ppara;* Mm00440939_m1), sterol regulatory element binding transcription factor 1 *(Srebf1;* Mm01138344_m1*)*, very low density lipoprotein receptor (*Vldlr*; Mm.PT.58.42530312 from IDT) and 18S ribosomal RNA (*Rn18s;* Mm03928990_g1) as the internal control gene. Reactions were run on a CFX96TM Real-Time System (Bio-Rad, Hercules, CA) according to the manufacturer’s instructions. Relative mRNA expression levels were calculated using the ΔΔCt method.

### Statistical analyses

When data were normally distributed, the student’s unpaired t-test was used to compare differences between groups; the nonparametric Mann–Whitney test was used for data that did not follow the Gaussian distribution. The two-way ANOVA with Sidak’s multiple comparisons test was used to compare differences among groups in serum lipids under fasting and postprandial conditions. The GraphPad Prism 6.0 software (GraphPad, San Diego, CA) was used to perform all statistical analyses. A p value of < 0.05 was considered to indicate statistical significance.

## Results

### Expression of human hepatic lipase increases animal weight and promotes hepatic fat accumulation

**Fig 1:**
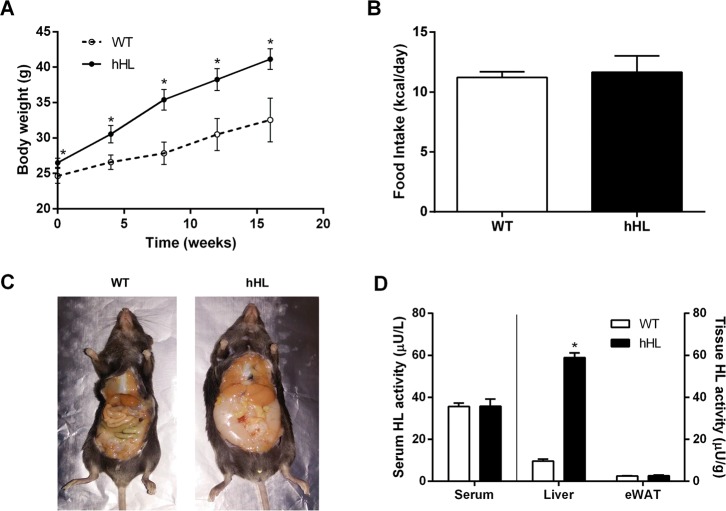
Overexpression of hHL in liver promotes diet-induced obesity and hepatomegaly. (A) Average monthly weight in WT and hHL transgenic mice. (B) Food intake per day and per mouse was monitored over 48 hours in the last week of the 16-week feeding regimen. (C) Representative adipose fat and liver from both WT and hHL transgenic mice. (D) Serum and tissue HL activities were measured using a radiolabeled glycerol tri[9,10-(n)-^3^H]oleate emulsion. For A, B, and D, values are mean ± SEM of 6 WT and 8 hHL transgenic mice and * indicates p <0.05 vs WT mice.

**Fig 2:**
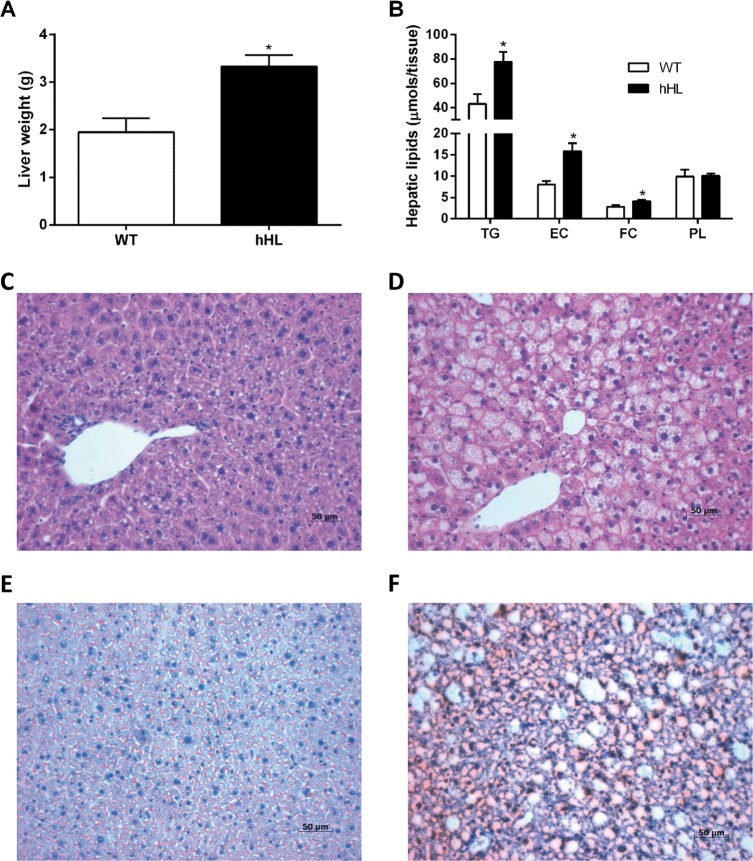
Human HL overexpression promotes hepatic steatosis. (A) Liver weights after the 16-week feeding experiment in hHL transgenic and WT mice. (B) Hepatic lipid levels of triglycerides (TG), esterified cholesterol (EC), free cholesterol (FC), and phospholipids (PL) were determined after lipid extraction with isopropyl alcohol-hexane. Values are mean ± SEM of 6 WT and 8 hHL transgenic mice and * indicates p <0.05 vs WT mice. Representative liver tissue sections from WT (C) and hHL transgenic mice (D) stained with H&E. Representative liver tissue sections from WT (E) and hHL transgenic mice (F) stained with Oil Red O. Livers from hHL transgenic mice showed severe macrovesicular steatosis with positive staining for neutral lipids.

To evaluate the effect of overexpressing hHL on diet-induced obesity and hepatic lipid accumulation, we carried out a 16-week study comparing WT and hHL transgenic mice after high-fat diet feeding, starting at eight to nine weeks of age. The body weight in the hHL-expressing animals was higher than that in the WT group throughout the 16 weeks (Fig 1A). However, no significant differences in food intake were found between mice of either genotype (Fig 1B). After the 16-week feeding regimen, the livers of the hHL-expressing mice were pale in color as compared to the healthy red color observed in the WT mice (Fig 1C). As expected, HL activity was mainly present in the livers of hHL transgenic mice, whereas WT and hHL-expressing mice showed similar basal levels of free HL in serum (Fig 1D). Consistent with the macroscopic changes found in the liver, the expression of hHL led to the development of steatosis as revealed by the increased liver weight (Fig 2A) and a higher hepatic lipid content, mainly by an increase of the triglyceride content (Fig 2B). These biochemical findings were confirmed by histochemical evidence of marked macrovesicular steatosis in hHL-expressing mice compared with the microvesicular steatosis pattern in WT mice (Fig 2C and 2D). The Oil Red O staining showed that WT livers displayed some isolated patchy red staining (Fig 2E). Conversely, the hHL transgenic mice livers showed widespread Oil Red O-positive macrovesicules (Fig 2F).

### Expression of human hepatic lipase promotes hepatic triglyceride lipogenesis without affecting triglyceride secretion or serum levels of triglycerides

**Fig 3:**
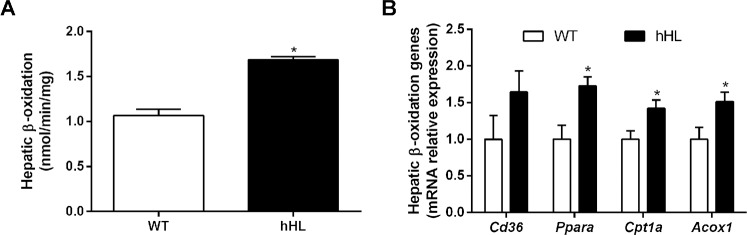
Transgenic mice overexpressing hHL showed increased fatty acid β-oxidation. (A) Liver fatty acid oxidation was determined as nmols of palmitoyl-CoA produced in both WT and hHL transgenic mice. B) Transcriptional expression of hepatic *Cd36* and the PPARα target genes *Cpt1a* and *Acox1*. Values are mean ± SEM of 6 individual animals per group and * indicates p <0.05 vs WT mice.

**Fig 4:**
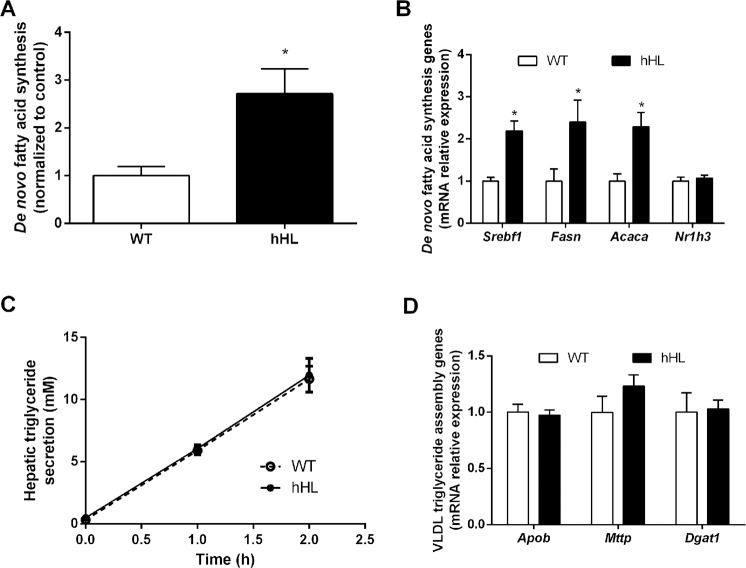
Expression of hHL promotes hepatic *de novo* lipogenesis without affecting triglyceride secretion. (A) *De novo* synthesis of fatty acids in livers of hHL transgenic and WT mice. The incorporation of ^3^H_2_O into fatty acids was determined after i.p. injection of 10 mCi/kg of ^3^H_2_O into each mouse. Values are mean ± SEM of 7 WT mice and 9 hHL transgenic mice. (B) Transcriptional expression of hepatic *Nr1h3* and the SREBP-1c target genes *Fasn* and *Acaca* (N = 6 mice/group). (C) *In vivo* triglyceride secretion rate in mice injected intravenously with Triton WR-1339. Mice were bled immediately prior to Triton WR-1339 injection and 60 and 120 min afterwards. Triglycerides were measured and their change with respect to the baseline result is shown (N = 3 mice/group). (D) Hepatic expression of *Apob*, *Mttp*, or *Dgat1* (N = 6 mice/group). * indicates p <0.05 vs WT mice.

To elucidate the causes of the increased hepatic triglyceride levels found in hHL transgenic mice, we studied whether these changes may be attributable to a defect in the rate of fatty acid oxidation or a higher rate of *de novo* triglyceride synthesis combined with impaired hepatic triglyceride secretion. However, the expression of hHL enhanced the hepatic rate of fatty acid oxidation by 60% (Fig 3A), which was associated with the upregulation of several key peroxisome proliferator-activated receptor (PPAR)α target genes (such as *Cpt1a* and *Acox1*) involved in fatty acid β-oxidation without affecting *Cd36* (Fig 3B). We then analyzed whether hHL expression affected *de novo* lipogenesis in the livers of transgenic mice. The *de novo* lipogenesis rate in the liver of transgenic mice was 2.5-fold higher than that of WT mice (Fig 4A). This increase was also associated with significant increases in mRNA levels of hepatic *Srebf1*, *Fasn*, and *Acaca*, but *Nr1h3* which express liver X receptor α was not altered by hHL expression (Fig 4B). In contrast, the rate of *in vivo* hepatic triglyceride secretion and the expression of key genes regulating very low density lipoprotein (VLDL) synthesis and assembly as well as hepatic triglyceride secretion were not altered significantly by hHL expression (Fig 4C and 4D). Importantly, the hepatic expression of *Apoa1*, *Ldlr* and *Vldlr* was upregulated in hHL transgenic mice, but not *Lcat* expression (S1 Fig). However, serum triglycerides and FFA under fasted or postprandial conditions were not significantly affected by the expression of hHL (Table 1).

**Table 1:** Serum lipids and lipoproteins in WT and hHL transgenic mice fed a Western diet for 16 weeks under fasting and postprandial conditions.

	Fasting conditions		Postprandial conditions	
	WT	hHL	WT	hHL
Serum triglycerides (mM)	0.25 ± 0.07	0.39 ± 0.03	0.72 ± 0.11^a^	0.50 ± 0.08
Serum FFA (mM)	1.04 ± 0.07	1.16 ± 0.06	0.62 ± 0.05 ^a^	0.67 ± 0.03 ^a^
Serum cholesterol (mM)	5.13 ± 0.28	4.87 ± 0.30	6.40 ± 0.25 ^a^	6.50 ± 0.47 ^a^
Serum phospholipids (mM)	4.12 ± 0.11	4.20 ± 0.11	5.32 ± 0.13 ^a^	4.73 ± 0.33
HDL cholesterol (mM)	4.42 ± 0.43	3.89 ± 0.22	5.07 ± 0.23	4.81 ± 0.33
HDL phospholipids (mM)	3.91 ± 0.10	3.90 ± 0.09	4.65 ± 0.14^a^	4.06 ± 0.28^b^

Values are mean ± SEM of 6 mice per group. Two-way ANOVA was used to compare differences among groups under fasting and postprandial conditions.

^a^p < 0.05 vs WT and

^b^p < 0.05 vs fasting conditions.

### Transgenic mice expressing human hepatic lipase exhibit more adiposity and upregulated adipose-specific LPL and FFA influx

**Fig 5:**
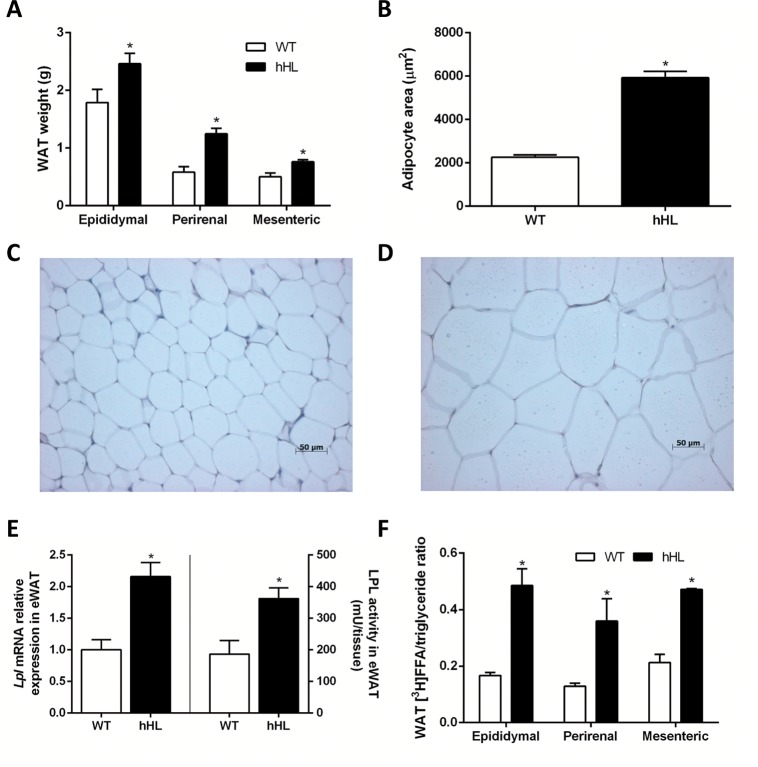
HL transgenic mice exhibit more adiposity and upregulated adipose LPL-mediated FFA influx. (A) Fat pad mass weights after the 16-week feeding experiment in hHL transgenic and WT mice. Each value represents the mean ± SEM of data from 6 WT mice and 8 hHL transgenic mice. (B) Quantitation of adipocyte size (N = 3 mice/group). Representative photomicrographs of H&E stained sections from eWAT of WT (C) and hHL transgenic mice (D). (E) Transcriptional expression of eWAT *Lpl* and tissue activity (N = 5 mice/group). (F) [^3^H]-triglyceride labeled triglyceride-rich lipoproteins were injected intravenously into WT and hHL transgenic mice under postprandial conditions and radiolabeled FFA and triglycerides were measured in WAT fat pads. Radiolabeled triglycerides + FFA in the collected fat depots did not differ between genotypes (0.92 ± 0.05 vs 0.80 ± 0.16% of the injected dose in WT and hHL transgenic mice, respectively). [^3^H]FFA/triglyceride ratios are shown (N = 3 mice/group). * indicates p <0.05 vs WT mice.

Transgenic mice displayed more adiposity than WT mice as shown in Fig 1C and as indicated by the amount of fat mass stored in the epididymal, mesenteric, and perirenal fat pads (Fig 5A). Consistent with the body adiposity, the size of hHL eWAT adipocytes was increased compared with that of WT adipocytes (Fig 5B), as shown histologically in Fig 5C and 5D. HL was expressed at low levels in the eWAT of both transgenic and WT mice (Fig 1D). In contrast, mice expressing hHL showed a higher expression of LPL in eWAT compared with that of WT mice (Fig 5E). To determine the fate of triglyceride-derived fatty acids in WT and hHL transgenic mice, we injected mouse TRL radiolabeled with [^3^H] triolein under postprandial conditions. We reasoned that the adipose fat pads tissues with higher expression of LPL would show the greatest increase in the triglyceride lipolysis and the uptake of radiolabeled FFA. Ten min after the injection of radiolabeled TRL, we found a notable increase of radiolabeled FFA into the different fat depots (Fig 5F), thereby suggesting that most of the label had undergone a significant lipolysis and uptake. Furthermore, the expression of hHL did not have any impact on *de novo* fatty acid synthesis in the adipose tissue, whereas the rate of fatty acid oxidation was very low (S2 Fig). Additionally, *Ldlr*, *Vldlr* and *Lipe* expression was not altered in hHL transgenic mice (0.59±0.11, 0.95±0.17 and 0.94±0.18, respectively, vs 1.00±0.24, 1.00±0.23 and 1,00±0.03, respectively, in WT mice).

## Discussion

Significant evidence indicates that hHL plays a key role on steatosis [10, 11]. Causality between HL deficiency and hepatic fat accumulation has been studied in genetically-modified models with divergent results [12, 13]. However, hHL is mainly bound to the cell surface of hepatocytes, whereas the mouse form is mainly found circulating in the bloodstream [15]. This study demonstrates, for the first time to our knowledge, that hHL promoted hepatic triglyceride accumulation mainly by increasing *de novo* triglyceride synthesis without affecting its secretion from the liver. By hydrolyzing lipoprotein-associated phospholipids and triglycerides, hHL may promote the uptake of FFA by the liver [25]. However, radiolabeled triglyceride-rich lipoprotein-derived FFAs were not differentially accumulated in the livers of hHL transgenic mice (S3 Fig). It should be noted that VLDL-triglyceride hydrolysis by HL is able to activate both PPARα and β/δ through generation of specific monounsaturated FFAs [26]. Therefore, it is possible that the hHL-mediated increase in PPAR target genes and the higher rate of β-oxidation could reduce the FFA available for triglyceride esterification, thus contributing to the unaltered triglyceride-rich lipoprotein-derived FFA levels that we found in the livers of our hHL transgenic mice. In contrast, hHL promoted hepatic *de novo* lipogenesis, which is considered a prominent abnormality in NAFLD development [27]. Of note, hepatic steatosis is usually associated to liver n-3 long-chain polyunsaturated fatty acid depletion, thereby leading to substantial enhancement in hepatic sterol regulatory element binding protein (SREBP)-1c/PPARα ratio that favors *de novo* lipogenesis over fatty acid β-oxidation [28, 29]. In line with these findings, we found that hHL upregulated the key enzymes involved in hepatic *de novo* lipogenesis, which are mainly regulated by the SREBP-1c encoded by the *Srebf1* gene [27]. Furthermore, PPARα may regulate positively the liver SREBP-1c target genes [30], as also occurred for *Ldlr* and *Vldlr* ([31, 32]). However, the ability of the liver to increase the secretion of triglycerides was rather reduced with respect to the increased hepatic *de novo* lipogenesis. Taken together, our data clearly indicate that the upregulation of the hepatic *de novo* lipogenesis is the major contributor to the stored liver triglycerides in mice overexpressing hHL. The low levels of VLDL and LDL and the high turnover of these lipoproteins in mice could have limited the impact of liver *Vldlr* and *Ldlr* upregulation on postprandial serum triglycerides of hHL transgenic mice [33, 34].

Rather surprisingly, hHL overexpression did not affect the HOMA-IR index and glucose tolerance (S4 Fig), despite the fact that it increased body weight and fat mass. The absence of any effect on glucose and insulin tolerance tests had been previously reported in HL-deficient mice [12]. Importantly, hHL had a limited effect on systemic FFA and triglyceride levels in our mice, as occurred in the HL-deficient mice fed the same Western-type diet [12]. These data contrast with those of an independent study carried out in HL-deficient mice fed a diet with a higher level of cholesterol that exhibited enhanced systemic inflammation, glucose intolerance, and higher FFA and triglyceride levels [13], thereby suggesting that the HL-mediated effect on glucose homeostasis requires an exacerbated inflammatory and dyslipidemic state, at least in mice. Of note, a direct association between hepatic steatosis and HL was found in NAFLD patients, even after controlling for insulin resistance [10], thus suggesting that insulin resistance seems to be secondary to the HL-mediated effects on steatosis.

Our data also demonstrated a stimulating effect of hHL on adipocyte LPL. This finding might suggest an increased release and uptake of FFA from circulating TRLs into adipocytes, which in turn might help to explain fat accumulation in these mice. The elevated proportion of radiolabeled FFA-to-triglycerides in adipose tissue of transgenic mice would support this hypothesis. It should be noted that hHL was not expressed in WAT, and neither did we find any alteration in the rate of β-oxidation or the regulation of *de novo* lipogenesis. In contrast, hHL is expressed in mouse adrenal cortex [35], and HL plays a key role in adrenal cholesterol delivery for steroid synthesis [36]. Although the effects of hHL on mouse adrenal response have not been investigated, it is possible that an enhanced adrenal corticosterone response [36] could upregulate LPL in eWAT [37, 38].

In the present study, the expression of hHL in transgenic mice had a limited impact on HDL lipid levels. The upregulation of liver *Apoa1* may have counterbalanced the HDL cholesterol-lowering effects of hHL. The absence of changes in HDL cholesterol levels had been previously reported in mice overexpressing HL at moderately high levels (up to 3-fold) [14, 16], but a contrast with the severe HDL deficiency showed when transgenic mice were fed a high-fat diet containing sodium cholate [35]. The discrepancy among these studies may be related to the different dietary regimens and the degree of HL expression, since the latter used a diet that induced proinflammatory and hepatotoxic effects and HL activity was 20-fold higher than that of non-transgenic mice [35].

In conclusion, our study demonstrates that hHL promoted hepatic steatosis in mice mainly by upregulating *de novo* lipogenesis without affecting hepatic triglyceride secretion and glucose homeostasis. The expression of hHL also promoted obesity and upregulated the adipose LPL-mediated FFA influx. Therefore, our data support the critical role of hHL in regulating hepatic triglyceride accumulation and its key influence on adipose tissue triglyceride homeostasis.

## Supporting information

S1 Fig Hepatic expression of *Apoa1*, *Ldlr* and *Vldlr* is upregulated in hHL transgenic mice. Transcriptional expression of hepatic *Apoa1*, *Lcat*, *Ldlr* and *Vldlr*. Values are mean ± SEM of 6 individual animals per group and * indicates p <0.05 vs WT mice.
(TIF)

S2 Fig Transgenic mice overexpressing hHL do not show any alteration in eWAT fatty acid β-oxidation and *de novo* fatty acid lipogenesis. (A) Epididymal WAT fatty acid oxidation was determined as nmols of palmitoyl-CoA produced in both WT and hHL transgenic mice under postprandial conditions. Values are mean ± SEM of 2 WT mice and 6 hHL transgenic mice. (B) The transcriptional expression of eWAT PPARα target genes *Cpt1a* and *Acox1* (N = 6 mice per group). (C) *De novo* synthesis of fatty acids in eWAT of hHL transgenic and WT mice. Mice were i.p. injected with 10 mCi/kg of ^3^H_2_O and sacrificed one hour later. The incorporation of ^3^H_2_O into fatty acids was determined after lipid extraction of the tissues with petroleum ether. Values are mean ± SEM of 7 WT mice and 9 hHL transgenic mice. (D) The transcriptional expression of SREBP-1c target genes *Fasn* and *Acaca* (N = 6 mice per group).
(TIF)

S3 Fig Triglyceride-rich lipoprotein-derived FFAs are not accumulated in the livers of hHL transgenic mice. [^3^H]-triglyceride labeled triglyceride-rich lipoproteins were injected intravenously into WT and hHL transgenic mice under postprandial conditions and radiolabeled FFA and triglycerides were measured in the livers of the same animals used in the Fig 5F.
(TIF)

S4 Fig Effects of hHL expression on insulin resistance. (A) Fasting serum glucose levels. (B) The homeostatic model assessment for insulin resistance (HOMA-IR). (C) Glucose kinetics following an intraperitoneal glucose tolerance test (IPGTT). (D) Area under the curve (AUC) of glucose after IPGTT. Values are mean ± SEM of 6 individual animals per group.
(TIF)
